# The enigmatic xenopsins

**DOI:** 10.7554/eLife.31781

**Published:** 2017-10-19

**Authors:** Detlev Arendt

**Affiliations:** 1 Developmental Biology UnitEuropean Molecular Biology LaboratoryHeidelbergGermany; 2 Centre for Organismal Studies (COS)University of HeidelbergHeidelbergGermany

**Keywords:** photoreceptor, opsin, evolution, eye, invertebrates, Leptochiton asellus, Other

## Abstract

A new member of the family of light-sensitive proteins called opsins has stirred up our view of photoreceptors.

**Related research article** Vöcking O, Kourtesis I, Tumu SC, Hausen H. 2017. Co-expression of xenopsin and rhabdomeric opsin in photoreceptors bearing microvilli and cilia. *eLife* **6**:e23435. doi: 10.7554/eLife.23435

How did animals develop the ability to detect light and see? The process began when a transmembrane protein evolved into an efficient light sensor called opsin (Feuda et al., 2012). Subsequently, a stunning diversity of opsins emerged in various animal phyla that set the stage for the evolution of eyes.

The two most-studied opsins are r-opsins, which are are found in rhabdomeric photoreceptor cells, and c-opsins, which are found in ciliary photoreceptor cells (Arendt et al., 2004). The r-opsins are commonly stored at the top of rhabdomeric cells in the folded cell membrane, called the rhabdom, which became the major light sensor in invertebrate eyes. In contrast, the c-opsins are transported into the cilium of ciliary photoreceptor cells, where the membrane is likewise folded to expand the light-sensitive surface. Ciliary photoreceptors are the main constituent of vertebrate eyes.

Besides the r- and c-opsins, which are well characterized, other opsins are now becoming the focus of attention. For example, tetraopsins are co-expressed with r-opsins in the eyes of segmented worms (Gühmann et al., 2015), and cnidopsins locate to the light-sensitive cilia of jellyfish eyes (Bielecki et al., 2014). The newest addition to the zoo of opsins, however, is an enigmatic group called the xenopsins (Ramirez et al., 2016). Now, in eLife, Harald Hausen and colleagues at the University of Bergen – including Oliver Vöcking, who is also at the University of Pittsburgh, as first author – report the results of a study that reveals more about how opsins evolved (Vöcking et al., 2017).

Vöcking et al. first present a molecular phylogeny, based on RNA sequence data, that distinguishes a total of ten distinct opsin families (Figure 1). Complementing this analysis, they then plot information about the introns (which are not present in the sequenced RNA molecules) of all available opsin genes, so that for the first time we can exhaustively compare how the opsin families are evolutionarily related with the structure of those genes. This analysis reveals distinctive intron patterns for the majority of the opsin families. Moreover, and rather strikingly, some introns are shared between families, suggesting that these families are more closely related: for example, the ‘anthozoan opsins II’ (only found in the polyps of anthozoans such as sea anemones and corals) and the ctenopsins (which are found in ctenophores, also known as comb jellies; Schnitzler et al., 2012) share one intron with each other and two introns each with c-opsins. This is in line with the results of the molecular phylogeny that likewise links the three families and indicates that the ctenopsins and the anthozoan opsins represent offshoots of the c-opsin branch. This is a huge step forward in our understanding of how c-opsin and ciliary photoreceptors evolved.

**Figure 1.**
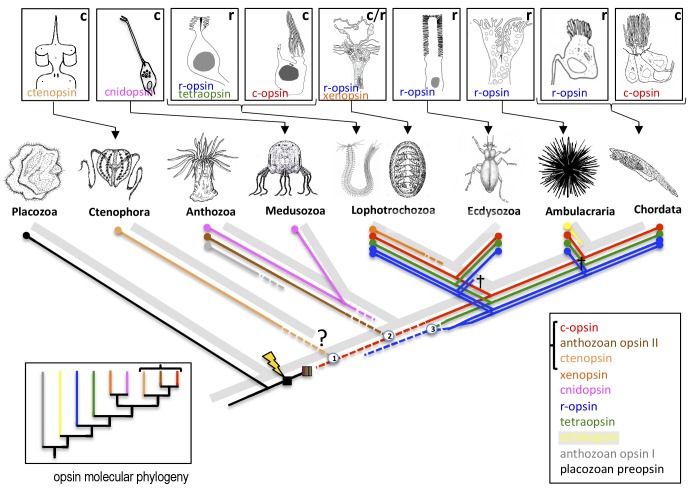
The evolution of opsins and photoreceptor cells. The animal evolutionary tree (light grey) shows the relationships between a number of different animal phyla; the question mark indicates the current uncertainty about when the ctenophores emerged. Different phyla have evolved their own photoreceptors (top; ‘r’ denotes rhabdomeric photoreceptors, ‘c’ denotes ciliary photoreceptors) that each use specific opsins to detect light (named in the panel of its respective photoreceptor). To investigate when these opsins and photoreceptors evolved, Vöcking et al. determined the molecular phylogeny of ten families of opsins (bottom left; colored lines correspond with the colored text in the bottom right box). Further confirmation that the bracketed opsins are closely related came from an analysis that shows that their genes share introns. Placing the opsin molecular phylogeny (colored lines in main image) on top of the animal evolutionary tree (light grey) suggests that if both are correct, certain opsins (such as the xenopsins) must have been independently lost from multiple phyla during evolution. Opsins first evolved after the divergence of the Placozoa (lightning bolt) and then went through an early period of diversification (striped box). Numbers in circles mark opsin family relationships that are supported by evidence from shared introns. Crosses indicate the loss of an opsin in a given lineage. Dashed lines indicate opsin relationships inferred from the intron comparison.

The position of the xenopsins, however, remains enigmatic. While the molecular phylogeny suggests they are closely related to the cnidopsins, the intron comparison reveals one shared intron with the r-opsins, which is insufficient to draw any conclusion. Even more puzzling, despite their apparent deep rooting in the opsin molecular phylogeny, the xenopsins are found only in the Lophotrochozoans (a superphylum that includes mollusks). This would imply that xenopsins have frequently been lost throughout animal evolution.

Complicating matters further, Vöcking et al. investigated the expression of xenopsin in the chiton, a marine mollusk. They found that xenopsin is co-expressed with r-opsins in the rhabdomeric photoreceptor cells of the larvae. They also found that these cells bear a cilium and express genes that help to localize opsins to cilia. Thus an r-opsin may be found in the rhabdom and a xenopsin in the cilium of the same photoreceptor, giving these cells a somewhat chimeric appearance.

To validate and explore this finding further, it will be necessary to test whether xenopsin and possibly other opsins indeed localize to the cilium and to determine the phototransduction cascades that the opsins trigger when they detect light. Only then will we understand how the diversity of the new opsins fits into the classical picture of rhabdomeric and ciliary photoreceptors.

Another challenge emerges from the molecular phylogeny. As it stands, this suggests that all opsin families outside of the c-opsin branch existed very early in animal evolution, even before the birth of the ctenophores (whose phylogenetic position is currently debated; see Simion et al., 2017). This is implausible for the xenopsins, especially so for the anthozoan and echinoderm opsins that only exist in one phylum, because it means they must have been lost in all other phyla. Of course, this paradox might also reflect the difficulty of using molecular phylogenies to resolve molecular interrelationships across animal phyla that separated more than 600 million years ago.
